# Remodeling of Gut Microbiome of Pakistani Expats in China After Ramadan Fasting

**DOI:** 10.1002/fsn3.70019

**Published:** 2025-02-26

**Authors:** Hafiz Arbab Sakandar, Feiyan Zhao, Jiahe Kang, Muhammad Nadeem Khan, Zhihong Sun

**Affiliations:** ^1^ Key Laboratory of Dairy Biotechnology and Engineering, Ministry of Education Inner Mongolia Agricultural University Hohhot China; ^2^ Key Laboratory of Dairy Products Processing, Ministry of Agriculture and Rural Affairs Inner Mongolia Agricultural University Hohhot China; ^3^ Inner Mongolia Key Laboratory of Dairy Biotechnology and Engineering Inner Mongolia Agricultural University Hohhot China; ^4^ Department of Microbiology, Faculty of Biological Sciences Quaid‐I‐Azam University Islamabad Pakistan

**Keywords:** gut microbiota, metabolic functionality, Ramadan, time‐restricted intermittent fasting

## Abstract

Time‐restricted intermittent fasting (TRIF) has gained popularity as an intervention for addressing overweight, obesity, and metabolic syndrome. It may influence the composition of the gut microbiome, potentially affecting various microbiome‐mediated functions in humans. However, limited studies have been conducted involving TRIF and microbiome on developing and underdeveloped populations. Here, we investigated the impact of TRIF/Ramadan fasting (16:8) on the changes of gut microbiome and functional profiling of microbial communities during and after the month of Ramadan in Pakistani Expats living in China. We observed substantial change in alpha diversity during TRIF; the changes in gut microbial structure by the end of TRIF were higher vis‐a‐vis in the beginning. Significant differences were observed among individuals; several bacteria (*Clostridium perfringens*, *Coprococcus comes* , and *Lactococcus lactis,* among others) were changed significantly (*p* < 0.05). Additionally, amino acid, carbohydrate, and energy metabolism; glycan biosynthesis; and metabolism of cofactors and vitamins were significantly affected by TRIF. Pyridoxamine, glutamate, citrulline, arachidonic acid, and short‐chain fatty acids showed substantial differences at different time points based on the predicted metabolic pathways. The preliminary results from this study demonstrate significant potential for elucidating the mechanisms underlying gut microbiome stability and enhancing the effectiveness of microbiome‐tailored interventions among the Pakistani populace to ameliorate metabolic disorders.

## Graphical abstract

Time‐restricted intermittent fasting (TRIF) significantly changed the microbial structure and compositions of Pakistani Expats living in China. TRIF mainly affected the gut microbial pathways of amino acid, carbohydrate, and energy metabolism and glycan biosynthesis metabolism of cofactors and vitamins.

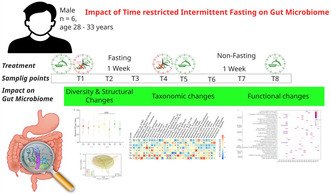

## 1 Introduction

Currently, there are around 2 billion followers of Islam worldwide who observe the month of Ramadan/month of time‐restricted intermittent fasting (TRIF) (9th lunar month of the Islamic calendar) every year, which affects the physiology of the body due to lifestyle alterations (de Cabo and Mattson 2019; Elmalti et al. 2023). During this month, it is an obligatory duty for all healthy Muslims to do dry intermittent fasting from sunrise (known as ‘Suhur’) to sunset (known as ‘Iftar’). Exceptions are granted to individuals who are unwell, pregnant, traveling, or exhibit underlying vulnerabilities that could potentially result in complications during fasting (Mohammed et al. 2023). Nonetheless, a considerable number of Muslims opt to observe their religious obligation and proceed with fasting during this period, despite dissuasion from medical professionals and treatment protocols (Su et al. 2021).

Mounting evidence suggests that the manipulation of a nutritionally balanced diet, achieved through caloric restriction, has the potential to postpone the onset and progression of diseases and promote a state of improved health and longevity in various organisms (Di Francesco et al. 2018). Intermittent fasting, a widely practiced diet‐related behavior, has demonstrated several health benefits. The American Heart Association reports that TRIF may lead to weight loss, mitigate insulin resistance, and decrease the risk of cardiometabolic disorders (St‐Onge et al. 2017; de Cabo and Mattson 2019). The contemporary trend of intermittent fasting stems from over a century's worth of scientific research demonstrating that a severe reduction of caloric intake, ranging from 20% to 40%, markedly enhances the lifespan of various animals, such as worms, flies, mice, rats, and rhesus monkeys, provided they receive sufficient nutrients. This outcome remains unparalleled among anti‐aging interventions. Moreover, numerous investigations involving fasting exhibit that severely restricted diets significantly diminish the occurrence of age‐related ailments, particularly cancer (Caprara 2018).

The underlying mechanisms mediating the effects of TRIF are still not fully understood, hampering the development of rational strategies to use intermittent fasting to treat disease and improve health. It has been posited that the conferring effects of fasting could be due to three major mechanisms, such as altered lifestyle, circadian biology, and modulation of the gut microbiome (Patterson and Sears 2017; Su et al. 2021), but the altered lifestyle and circadian biology have a great influence on gut microbiota. So, the gut microbiome plays an important role in the mediation of TRIF on health. However, the effects of intermittent fasting on the human microbiome are still obscure. Serval studies have reported the beneficial effects of Ramadan on intestinal microorganisms, such as increasing the α‐diversity, increasing beneficial bacteria reducing conditional pathogens in the gut (Ozkul, Yalinay, and Karakan 2020; Su et al. 2021), but Jo et al. reported that the decrease in the levels of short chain fatty acids (SCFAs) and beneficial bacteria during Ramadan, along with the increased microbial diversity post‐Ramadan, suggests that the daily diet during Ramadan may not provide adequate nutrients to maintain robust gut microbiota (Jo et al. 2023). Nevertheless, there are many factors that affect the intestinal flora, and it is normal that there are conflicts between the results. At the same time, all these studies take samples at a fixed time (usually at the beginning and ending of the Ramadan), and we can only get some information about the flora that has been greatly affected by Ramadan, and some microorganisms that have changed little and have been rapidly affected by changes in life and eating patterns have been ignored. Therefore, a dynamic sampling experiment was designed in this study, and samples were collected dynamically during and after Ramadan, aiming at analyzing the dynamic changes of the diversity, composition, function, and predicted metabolites during and after Ramadan.

## 2 Materials and Methods

### 2.1 Study Design

Ramadan was held from April 3rd to May 2nd in 2022. This study included six healthy Pakistani males (aged 28–33 years old) who lived in Hohhot (Inner Mongolia Autonomous Region, China) for a long time and participated in Ramadan all the time and strictly carried it out. During Ramadan, all volunteers strictly abide by injunctions. TRIF was involved in eating within an eight‐hour window and fasting for the remaining 16 h (16:8). The fecal samples were collected on the tenth (T1), eighteenth (T2), 30th day (T3) of Ramadan and the first (T4), second (T5), third (T6), seventh (T7), and 30th day (T8) after Ramadan. All subjects participating in the study were free of any gastric or metabolic conditions and had abstained from taking antibiotics for 6 months before and during the experiment. Furthermore, participants were instructed to maintain their typical dietary and lifestyle habits but to avoid any other products or foods advertised or marketed as probiotics during this study.

### 2.2 DNA Extraction and Shotgun Metagenomic Sequencing

QIAamp Fast DNA Stool Mini Kit was used to extract metagenomic DNA from stool samples. Afterwards, we measured the completeness, concentration, and purity of DNA using Qubit 2.0 Flurometer, NanoDrop One, and 1% agarose gel electrophoresis (Zhang et al. 2016). The DNA with optical density (OD) 260/280 between 1.8 and 2.0, concentration > 20 ng/μL, and little fragmented was judged to be qualified. The DNA sequence library was conducted by NEBNext UltraDNA Library Preparation Kit with 1 μg of metagenomic DNA as recommended by the manufacturer.

To summarize, metagenomic DNA was fragmented into 350 bp using sonication. The ends of each DNA fragment were polished, A‐tailed, and then ligated to full‐length aptamers to enable Illumina sequencing and subsequent PCR amplification. Following this, PCR products were purified using the AMPure XP system, and the size distribution of the library was evaluated using the Agilent 2100 Bioanalyzer. The Illumina NovaSeq 6000 (Illumina Inc. California, USA) sequencer was used for metagenomic sequencing.

### 2.3 Bioinformatics of Metagenomic Data

All samples generated 365 Gb of paired‐end bases (7.61 ± 1.61 Gb raw metagenomic data per sample). Low‐quality raw data and host DNA sequences were removed by Trimmomatic (v.0.3.9) and Bowtie2 (v.2.4.4) in KneadData (Kharofa et al. 2023). Finally, a total of 358 Gb of clean bases were used for downstream analysis (7.47 ± 1.58 Gb clean metagenomic bases per sample). MetaPhlan3 software was used to annotate and classify the metagenomic species. HUMAnN3.0 was used for functional genes and metabolic pathway annotation of the final reads (Shumyatsky et al. 2023). Metabonomic predictions were based on gene abundances by the MelonnPan‐predict workflow.

### 2.4 Statistical Analysis

All statistical analyses were carried out in R software (www.r‐project.org). The alpha diversity of gut microbiota was calculated by the vegan package (v.2.5.7) in R. Principal coordinates analysis (PCoA) based on Bray‐Curtis distance was performed to evaluate overall changes in the gut microbiota community structure calculated by the ggpubr package (v.0.4.0). The difference in the structure of gut microbiota in all sampling points were calculated by analysis of similarities (Anosim test; 999 permutations), and *p* < 0.05 was considered significant. Comparisons among groups were used Wilcoxon tests (cut‐off level: *p* < 0.05). Spearman's correlation networks of bacterial genera were constructed by Cytoscape (v3.5.1), with cut‐off levels of |*r*| > 0.9 and *p* < 0.05. Data were visualized using R and Adobe Illustrator (Adobe Inc., California, USA).

## 3 Results

### 3.1 Changes in Gut Microbiota Diversity

The study involved six male participants aged 28–33 who fasted for 30 days during the month of Ramadan/ TRIF. The composition of their gut microbiota in response to TRIF was analyzed using metagenomic sequencing, both during Ramadan and up to 30 days after Ramadan.

**FIGURE 1:**
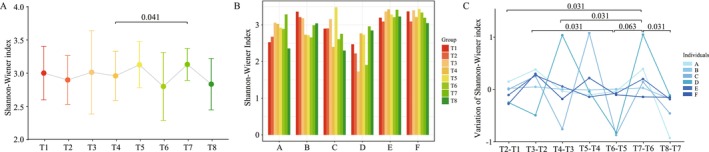
Shannon‐Wiener index was used to show the diversity of gut microbiota with the progression of TRIF. The Shannon‐Wiener index in different time points (A) and different participants (B); (C) the changed Shannon‐Wiener index between two adjacent time points during the trial for every participant. Wilcoxon test was used to evaluate statistical differences, and *p* < 0.05 was considered significant.

The analysis revealed that TRIF had an impact on the diversity of the gut microbiota. The Shannon‐Wiener index showed that the diversity of the gut microbiota started to change during Ramadan and was observed as slightly high at the end of the fasting period. However, when compared to later stages, the diversity was found to be significantly increased (*p* = 0.041), as shown in Figure 1A.

At the individual level, it was found that there was variation in the gut microbiota between participants, as shown in Figure 1B. However, there was a consistent trend that the diversity of the gut microbiota changed significantly in response to TRIF, and the changes were more pronounced 1 week after the end of the fasting period (T7), as shown in Figure 1C.

### 3.2 Changes in Gut Microbiota Structure

**FIGURE 2:**
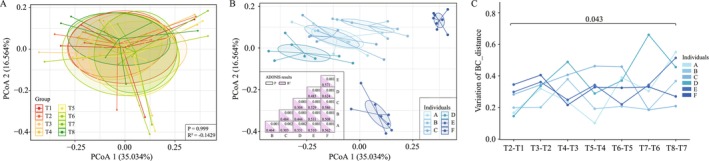
Principal coordinates analysis (PCoA) based on Bray‐curtis distance was used to show the structure of gut microbiota during the study. The structure of gut microbiota at different time points (A) and different participants (B) during the study; (C) the changed distance between two adjacent time points during the study for every participant. *p* < 0.05 was considered significant.

The study also found that TRIF had an impact on the structure of the gut microbiota of the subjects. When the gut microbiota was clustered using the Bray‐Curtis approach, it was observed that there was no significant effect (*p* > 0.05) of TRIF on the overall structure of the gut microbiota, as shown in Figure 2A.

However, when the gut microbiota was evaluated at the individual level, significant variation (*p* > 0.05) was observed among the subjects, as shown in Figure 2B. To further investigate this variation, the changes in Bray‐Curtis distance were calculated for each individual with respect to time interval, and it was observed that the changed structure of the gut microbiota between 30 days (T8) and 7 days (T7) after Ramadan was significantly higher than during Ramadan (*p* = 0.043), as shown in Figure 2C.

This suggests that the effect of TRIF on the gut microbiota may become more pronounced after Ramadan ends, and that individual variation may also be more pronounced during this time.

### 3.3 Specific Taxonomic Changes

**FIGURE 3:**
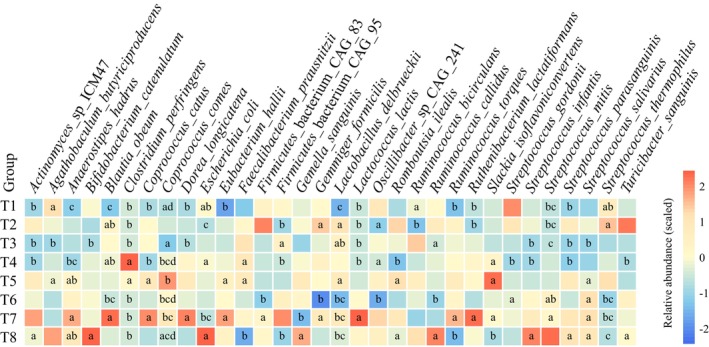
Significantly different abundant species during TRIF. The color scale illustrates the relative abundance; the intensity of red and blue represents higher and lower relative abundances, respectively. The wilcoxon test was used to evaluate statistical differences; *p* < 0.05 was considered significant, the significance was indicated by the letter‐based method, only different letters indicate that there are significant differences between groups; same letter or no mark indicate no significant difference among groups.

Although the composition of gut microbiome varies gently, the TRIF had a significant effect on the composition of gut microbiota at the species level. During the TRIF trial, it was observed that the relative abundance of certain bacterial species, including *Clostridium perfringens, Coprococcus comes, Escherichia coli*, and *Firmicutes bacterium* CAG95 were significantly affected either immediately after the TRIF started or during its course. Briefly, *C. perfringens* increased in response to TRIF while *E. coli* and *Firmicutes* decreased in response to TRIF (Figure 3). Following completion of the TRIF, it was observed that the relative abundance of certain bacterial species, including *Actinomyces* sp. ICM47, *Bifidobacterium catenulatum* , *Faecalibacterium prausnitzii, Firmicutes bacterium* CAG95, *Ruminococcus callidus, Slackia isoflavoniconvertens, Streptococcus infantis, Streptococcus parasanguinis, Streptococcus salivarius*, and *Streptococcus thermophilus*, were significantly affected, with changes becoming apparent at the second or third time points after the study ended. Briefly, *Actinomyces* sp. ICM47, *B. catenulatum, F*. *prausnitzii* , *F. bacterium* CAG95, *S. soflavoniconvertens, S. infantis, S. parasanguinis*, and *S. salivarius* were increased in response to TRIF. The species *R. callidus* and *S. thermophilus* decreased in response to TRIF (Figure 3).

**FIGURE 4:**
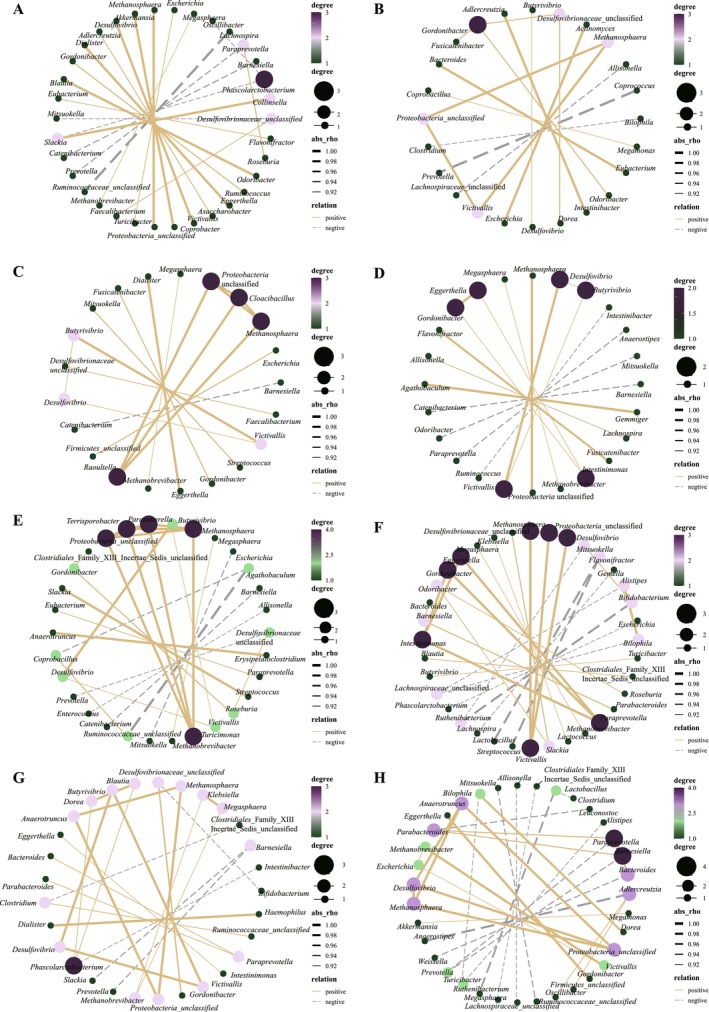
Genus‐level co‐occurrence networks during the trial (A–H represent T1‐T8, respectively). Gray and yellow lines represent positive and negative correlations; thick and thin lines represent correlation strength corresponding to Spearman's Rho. The co‐occurrence networks were more and more weakened with the process of TRIF; however, co‐occurrence networks were stronger after the TRIF, indicating the higher effect of TRIF on co‐occurrence networks between bacteria rather than bacterial diversity and structure.

To find the relationship between different genera of bacteria over time in the context of TRIF, genus‐level co‐occurrence networks were constructed. It has been observed that the co‐occurrence networks became progressively weaker during the TRIF trial, indicating that TRIF had a strong effect on the interactions between different genera of bacteria in the samples. However, after the TRIF trial was completed, the co‐occurrence networks gradually became more tightly connected. This suggests that the impact of TRIF was more pronounced on the specific interactions between the different genera of bacteria, rather than on the overall diversity and structure of the bacterial community. In other words, TRIF had a more targeted effect on the interactions between specific groups of bacteria rather than on the overall composition of the bacterial community (Figure 4).

This finding is important because it provides insights into how TRIF can affect the interactions between different bacteria in a microbial community. Understanding the mechanisms by which TRIF impacts bacterial interactions can have important implications for the development of new treatments for microbiome‐related disorders.

### 3.4 Changes in Functional Profiling

**FIGURE 5:**
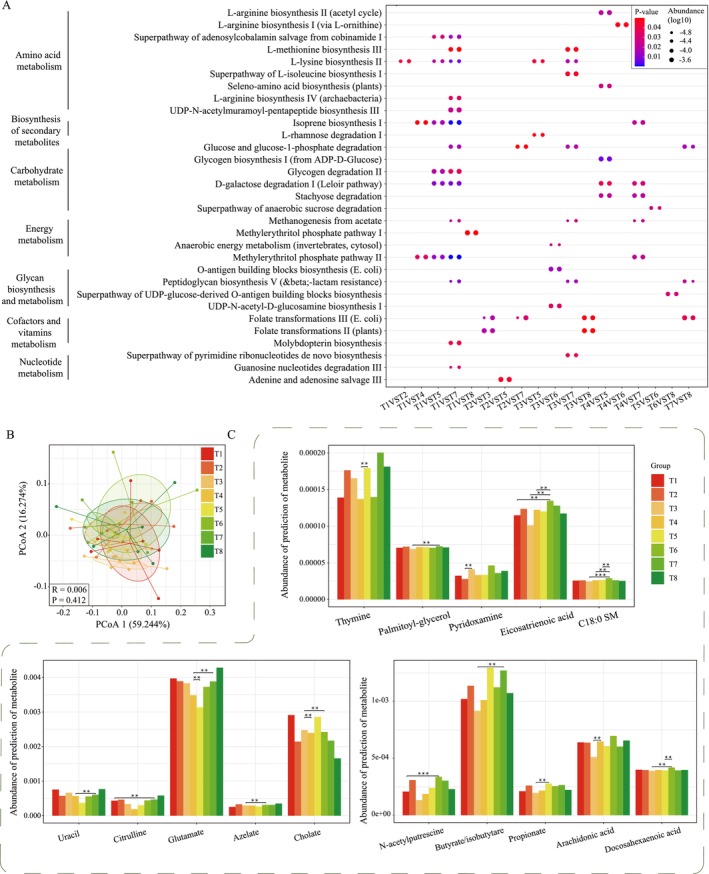
Changes in gut microbial pathways and predicted gut bioactive metabolome. (A) changed abundance of gut microbial pathways during TRIF; (B) Principal coordinates analysis (PCoA) score plots showing changes in the predicted gut bioactive metabolome at different time points during the trial; (C) bar charts comparing abundances of predicted differential bioactive metabolites that were responsive to the TRIF.

The changes of gut microbiome inevitably affect the alterations of functional pathways and metabolites, so we also explored the changes of functional pathways and predicted metabolites during and after TRIF. The functional pathways of amino acid metabolism, biosynthesis of secondary metabolites, carbohydrate metabolism, energy metabolism, glycan biosynthesis and metabolism, metabolism of cofactors and vitamins, and nucleotide metabolism were all changed during TRIF (Figure 5A). Specifically, we found that T1 and T7 were the most differentiated pathways (14 in total), followed by T3 and T7 (7 in total). This suggests that changes in the pathways of action of the gut microbiota may be a relatively slow process. However, when comparing T5, we found that there were differences between T5 and several sampling points during Ramadan and the first day after Ramadan (T4), and the differences were mainly concentrated in amino acid metabolism and carbohydrate metabolism; for example, L‐lysine biosynthesis II and L‐rhamnose degradation I pathways were significantly up‐regulated after TRIF. It has been reported that L‐rhamnose‐associated molecules are valuable for tumor immunotherapy and can improve organ damage and mortality. Gut microbiota‐originated metabolites were predicted by MelonnPan, a total of 80 metabolites were identified. The changes of predicted metabolites were similar as gut microbiome, which were samples from the same person that were more concentrated, not at the same time (Figure 5B). However, we also found some trends; that is, all the differential metabolites were concentrated at the comparison of TRIF (T1‐T3) and after TRIF (T4‐T8), such as the levels of pyridoxamine, glutamate, citrulline, arachidonic acid, and SCFAs (such as butyrate and propionate), and there is no significant change during TRIF (T1‐T3, Figure 5C). Moreover, the most differential metabolites were concentrated at T7 and other time points, which is consistent with the changes of metabolic pathways (Figure 5C).

## 4 Discussion

The gut microbiota plays an important role in health and disease, and it is influenced by many factors, including dietary habits and lifestyle (Bhattarai and Janaswamy 2022). Ramadan fasting is a month‐long religious practice observed by Muslims around the globe that involves abstinence from food and drink from dawn to dusk (Rashed 1992). It involves significant changes in dietary habits, with fasting during the daytime and meals consumed before sunrise and after sunset. It has been shown to have various health benefits, including improved insulin sensitivity, reduced inflammation, and enhanced autophagy (Abdeen and Elinav 2021). However, its effect on the gut microbiota is not well explored, especially in the Pakistani populace. Given this, the current study was conducted to investigate the impact of Ramadan fasting/TRIF on the structure and composition of the gut microbiota of the study participants from Pakistan.

Studies have reported that TRIF significantly changes the diversity of gut microbiota (Su et al. 2021; Khan et al. 2022). The same results were found in the present study; in the comparison of diversity during TRIF, we found that there were substantial differences among individuals, as in some individuals it increased the diversity and in others it decreased, which may be related to the physiological characteristics and living habits (Khan et al. 2022). However, we still found some trends; for example, with the progress of TRIF, the gut bacterial diversity of most volunteers increased, and the gut bacterial structure also changed greatly, such changes were partially explained by the meals eaten during TRIF containing more foods rich in carbohydrates and fiber, such as soups, porridges, legumes, and whole grains (Shadman et al. 2014; Hassanein et al. 2021). Su et al. (2021) found that the structure of gut microbial flora can be restored to the previous level after TRIF, but we found a different result that was the overall change of intestinal flora structure of subjects was not significant during TRIF but compared with the changes of each volunteer during TRIF, the change of gut microbial flora increased significantly between 1 week and 1 month after TRIF, which may be related to the sampling time, firstly, we did not collect the samples before TRIF; secondly, the interval between last two time points (T7 and T8) was too long. But in any case, we have seen the dynamic changes of intestinal flora after TRIF, that is, the diversity drops rapidly and the intestinal flora structure gradually recovers rather than rapidly, which is a phenomenon that has not been reported in previous studies.

Similarly, when the effect of TRIF was observed on specific taxonomic features of gut microbiota, several genera and their species appeared to be significantly changed. In the current study, the abundance of *Blautia obeum* and *Lactobacillus delbrueckii* was increased, while the abundance of *Agathobaculum butyriciproducens*, *Escherichia coli,* and *Ruminococcus bicirculans* was decreased significantly in response to TRIF. *B. obeum* and *L. delbrueckii* are often considered as beneficial bacteria. *B*. obeum was considered to generate high levels of propionate (Reichardt et al. 2014), and the colonization by representative *B. obeum* could protect from enteric virus infection, inducing type I interferon (IFN‐I) responses in macrophages via the MAVS‐IRF3‐IFNAR signaling pathway (Wang et al. 2023); many strains of *L. delbrueckii* were reported as a probiotic added in the yogurt, and many studies reported that the abundance of *L. delbrueckii* is positively correlated with the improvement of some health index (Mogna et al. 2016; Wu et al. 2024). Therefore, it could be inferred that TRIF could be effective in enhancing the abundance of beneficial bacteria in the gut. Other studies have also reported that TRIF can decrease the number of pathogenic bacteria in the gut. A study conducted on Pakistani and Chinese populations showed that Ramadan fasting reduced the members of the *Coprococcus* genera, while another study conducted on the Pakistani population has linked low relative abundance of *E. coli* and *Campylobacter* with Ramadan fasting (Su et al. 2021; Khan et al. 2022). After the end of Ramadan, we found that the *C. perfringens* and *Firmicutes bacteria* CAG 95, and with the recovery of normal work, rest, and eating, *C. perfringens* decreased significantly, while *G. sanguinis* , *L. lactis* , *R. ilealis*, *R. torques* , *S. salivarius* , and *S. parasanguinis* increased. *C. perfringens* is a pathogenic bacterium in intestine, the increased abundance may be a temporary disorder of intestinal flora caused by rapid changes in diet and living habits. Other significantly changed bacteria could also have specific functions such as production of SCFAs, but there were no specific changes among time points, which may be due to the early effect of TRIF on some bacteria and the late effect on others being proved to be transient and permutant bacteria, respectively (Erkosar and Leulier 2014). Additionally, the co‐occurrence networks became progressively weaker during the TRIF, and the networks became increasingly tight after the TRIF, which suggests that the impact of TRIF on the gut microbiota was not limited to changes in overall bacterial diversity and structure but also included changes in the way different bacterial genera interact with each other. This finding highlights the importance of considering not just the individual bacterial species present in the gut but also the interactions between them.

Along with taxonomic changes, predicted gut microbiota‐associated metabolism and metabolites were also observed to be modulated due to TRIF. Although research on this subject is scarce, the few studies that are on this specific topic present similar results where gut metabolites are modulated by TRIF (Guo et al. 2021; Maifeld et al. 2021; Mohammadzadeh et al. 2021). In the present study, we also found some functional pathways that changed during the trial were consistent with previous studies, such as amino acid metabolism, carbohydrate metabolism, and energy metabolism. TRIF has shown an increase in the catabolism of amino acids, leading to increased release of amino acids into circulation and subsequent use as an energy source (Cahill Jr 2006). The increased catabolism of L‐methionine, L‐lysine, and L‐isoleucine during the first week of the trial may be a result of this increased demand for energy. Conversely, the increased biosynthesis of L‐arginine and selenoamino acids during the last week of the TRIF may be a compensatory response to the decreased availability of dietary sources of these amino acids during TRIF (Nowak et al. 2018); TRIF has shown an increase in glucose production via glycogenolysis and gluconeogenesis, while decreasing glucose uptake and utilization in peripheral tissues (Rizza 2010). The observed changes in glycogen metabolism and sucrose degradation during the TRIF trial may reflect these changes in glucose homeostasis. Additionally, the observed changes in D‐galactose degradation may be a result of altered galactose metabolism during TRIF (Ma, Robinson, and Towle 2006); TRIF has also shown an increase in the utilization of alternative energy sources, such as ketone bodies and fatty acids, while decreasing reliance on glucose as an energy source (Evans, Cogan, and Egan 2017). The observed changes in methanogenesis from acetate and the methylerythritol phosphate pathway during the first week of the trial may reflect these changes in energy metabolism. Additionally, the observed changes in anaerobic energy metabolism during the third week of the TRIF may be a result of the switch to alternative energy sources.

Meanwhile, we also found some functional pathways changed during TRIF that were rarely reported in previous studies, such as the changes of isoprene biosynthesis and L‐rhamnose degradation that may alter lipid metabolism and increase oxidative stress during TRIF (Elahi et al. 2009). UDP‐N‐acetyl‐D‐glucosamine biosynthesis I, another glycan biosynthetic pathway, was found to be significantly changed during the third week of the TRIF. UDP‐N‐acetyl‐D‐glucosamine is a precursor for the synthesis of several glycan molecules, including peptidoglycan, lipopolysaccharides, and glycans of glycoproteins. The alteration of this biosynthetic pathway could have significant downstream effects on glycan synthesis and cell wall structure. Additionally, we found an interesting result, that is, on the 1 week after the end of TRIF, the functional pathway changed the most, suggesting that it may be a turning point in the recovery of intestinal flora, but this still needs more sampling time and a big sample size to prove.

In the present study, changes in predicted metabolism and metabolites over different time points can be correlated with microbiota stability and taxonomic changes (Vrieze et al. 2010; Erkosar and Leulier 2014). A small sample size is the limitation of this study, while evaluating the impact of TRIF weekly and comparing it with the post Ramadan microbiome are the strengths of the current study.

## 5 Conclusion

In summary, it can be concluded from the current study that despite many factors, such as geographical location, host's genetics, and health status, TRIF modulates the structure, composition, and gut metabolic profile of gut microbiota. The effect of TRIF is also personal and mostly normalizes the gut microbiota. TRIF could be implicated for various conditions, including obesity, type 2 diabetes, hypertension, and cardiovascular risk factors. Moreover, it alters the body's metabolic state from glucose oxidation to lipid oxidation, resulting in the reprogramming of metabolism, which has been disrupted by the contemporary Western diet. Additionally, determining whether TRIF can significantly enhance health or increase human lifespan would require numerous volunteers to maintain a fasting routine over a longer time, displaying extraordinary discipline.

## Author Contributions

**Hafiz Arbab Sakandar:** conceptualization (equal), data curation (equal), funding acquisition (equal), methodology (equal), project administration (equal). **Feiyan Zhao:** data curation (equal), formal analysis (equal), software (equal), visualization (equal). **Jiahe Kang:** software (equal), visualization (equal). **Muhammad Nadeem Khan:** writing – review and editing (equal). **Zhihong Sun:** conceptualization (equal), funding acquisition (equal), methodology (equal).

## Ethics Statement

The study protocol was approved by the Ethical Committee of the Inner Mongolia Agricultural University, Hohhot, China (Approval number A202205005). All procedures performed in studies involving human participants were in accordance with the ethical standards of the institutional and/or national research committee and with the 1964 Helsinki declaration and its later amendments or comparable ethical standards. Written informed consent was obtained from all subjects.

## Conflicts of Interest

The authors declare no conflicts of interest.

## Data Availability

The sequence dataset was deposited in the National Genomics Data Center (CNCB, https://ngdc.cncb.ac.cn/, accession number CRA013099).
